# Replication of *Salmonella enterica* serovar Typhimurium in RAW264.7 Phagocytes Correlates With Hypoxia and Lack of iNOS Expression

**DOI:** 10.3389/fcimb.2020.537782

**Published:** 2020-11-30

**Authors:** Marie Wrande, Kim Vestö, Speranta Puiac Banesaru, Naeem Anwar, Johan Nordfjell, Lifeng Liu, Gerald M. McInerney, Mikael Rhen

**Affiliations:** ^1^ Department of Microbiology, Tumor and Cell Biology, Karolinska Institutet, Stockholm, Sweden; ^2^ Department of Neuroscience, Karolinska Institutet, Stockholm, Sweden; ^3^ Department of Molecular Biology, Umeå University, Umeå, Sweden

**Keywords:** *Salmonella enterica*, hypoxia, iNOS, macrophages, bacterial infection

## Abstract

*Salmonella* infection associates with tissue hypoxia, while inducible nitric oxide synthase (iNOS), relying for its activity on molecular oxygen, stands as a central host defence measure in murine salmonellosis. Here, we have detailed hypoxia and iNOS responses of murine macrophage-like RAW264.7 cells upon infection with *Salmonella enterica* serovar Typhimurium. We noted that only a proportion of the infected RAW264.7 cells became hypoxic or expressed iNOS. Heavily infected cells became hypoxic, while in parallel such cells tended not to express iNOS. While a proportion of the infected RAW264.7 cells revealed shutdown of protein synthesis, this was only detectable after 12 h post infection and after iNOS expression was induced in the cell culture. Our data implicate an intrinsic heterogeneity with regard to hypoxia and iNOS expression in a cell culture-based infection setting.

## Introduction

Upon local hypoxia, the affected tissue is deprived of normal oxygen levels. Hypoxia occur in stroke patients (Roffe, 2002), within solid tumors (Sun et al., 2011) and within infectious granulomas (Rustad et al., 2009). As oxygen levels decrease or cells are exposed to various pathogens, the transcription factor hypoxia inducible factor, HIF, becomes activated (Hellwig-Bürgel et al., 2005; Peyssonnaux et al., 2005; Liu et al., 2010). The effects of hypoxia during infection could be many. Various innate immune responses are activated, such as cytokine expression and production of inducible nitric oxide synthase (iNOS) (Jung et al., 2000; Hellwig-Bürgel et al., 2005). In addition, hypoxia is reported to increase the phagocytic propensity of macrophages (Anand et al., 2007) and dampen replication of *Mycobacterium tuberculosis* in granulomas (Rustad et al., 2009; Schaffer and Taylor, 2015) while essential virulence functions of *Pseudomonas aeruginosa* (Scotti et al., 2014) and the intestinal pathogen *Shigella flexneri* are regulated by oxygen tension (Marteyn et al., 2010).

Macrophages play a central role in the host defence against invading pathogens, such as *M. tuberculosis* (Rustad et al., 2009) and *Salmonella enterica* (Mastroeni et al., 2000; Monack et al., 2004). One major macrophage antimicrobial feature is the production of nitric oxide (NO) through iNOS activity (Eriksson et al., 2000; Mastroeni et al., 2000). NO can further combine with superoxide radicals to form peroxynitrite and/or nitrosothiols, nitrogen dioxide and other nitrosating species (Xia and Zweier, 1997), with the capacity to cause damage to a variety of biological constituents (Rhen, 2019), as well as the potential killing of both pathogen and host cell (Chandra et al., 2000; Zhang et al., 2005).

*Salmonella* sp. are facultative intracellular Gram-negative bacteria that can invade vertebrate hosts through the intestinal mucosa and replicate in macrophages. *Salmonella’*s ability to cause disease relies on specific virulence factors, many of which are coded for by horizontally acquired genetic elements termed ‘*Salmonella* pathogenicity islands’ (SPIs) (Hansen-Wester and Hensel, 2001; Monack et al., 2004; Mastroeni and Grant, 2011). In the murine salmonellosis model, SPI1 is needed for invading non-phagocytic cells in the small intestine (Hansen-Wester and Hensel, 2001; Galan, 2001), while SPI2 is crucial for the intracellular phase of infection (Hensel, 2000; Figueira and Holden, 2012). By inducing the SPI2 genes, the bacteria are able to create and replicate in the *Salmonella*-containing vacuole (SCV) inside host cells.

During *Salmonella* pathogenesis, there is a complex interplay between NO and SPI2 activity. While SPI2 seems to protect the intracellular bacteria against NO, high levels of NO can down-regulate SPI2 expression (Chakravortty et al., 2002; McCollister et al., 2005; Bourret et al., 2009). Furthermore, nitrosylation of a specific cysteine residue in the SPI2 gene activator protein SsrB adds to virulence (Husain et al., 2010). In addition, the NirC nitrite transporter of *S*. Typhimurium leads to down regulation of IFN-γ induced iNOS expression and NO production (Das et al., 2009).

Recent studies on both eukaryotic and prokaryotic cells imply cell heterogeneity in selected settings, such as tumor tissue (Tellez-Gabriel et al., 2016) or among macrophages in particular (Martinez and Gordon, 2014). In mice infected with *Salmonella*, a regional iNOS expression in the spleen influences the abundance of local bacterial populations and that infected red pulp macrophages expressing low levels of iNOS are found in the close vicinity of highly iNOS positive cells (Burton et al., 2014). For *S*. Typhimurium, the bacterium is also believed to display heterogeneity in terms of SPI1 expression and host adaptation upon infection (Hautefort et al., 2003; Diard et al., 2013) or when contained in biofilms (Choong et al., 2016).

Murine macrophage cell lines are commonly used as *in vitro* hosts for *S*. Typhimurium intracellular replication and transcriptomic analyses. Most studies dealing with the iNOS response during infections have looked at total populations of cultured cells or whole tissue. While details of these responses on the single cell level point to heterogeneity among host cells (Saliba et al., 2017), even in *Salmonella*-infected tissue (Pham et al., 2020), a number of facets are still to be verified and detailed. In this study, we wanted to understand the details of hypoxia and iNOS responses in RAW264.7 cells infected with *S*. Typhimurium at the single cell level using fluorescence microscopy and flow cytometry. Our main findings were that *S*. Typhimurium replicate preferentially in iNOS negative macrophages and that extensive intracellular replication leads to hypoxia.

## Materials and Methods

### Bacterial Strains and Growth Conditions

The bacterial strains used throughout the study were *Salmonella* *enterica* serovar Typhimurium strain 14028. For some experiments, the bacteria contained plasmids expressing green fluorescent protein (GFP). The plasmids were under the control of L-arabinose inducible promoters, contained ampicillin resistance genes, and were a kind gift from Professor Oliver Pabst (Aachen University, Germany). Alternatively, the bacteria were chromosomally marked with a gene expressing yellow fluorescent protein (*galK*::YFP-*bla*). The bacteria were cultured at 37°C on Luria-Bertani (LB) agar plates supplemented when needed with 100 μg/ml of ampicillin or 50 μg/ml of chloramphenicol both purchased from Sigma (Sigma, St. Louis, MO, USA).

### Cell Culture

The murine RAW264.7 macrophage-like cell line (ATCC) was cultured in RPMI 1640 medium (Gibco, Paisley, UK), supplemented with 10% fetal bovine serum (FBS) (Gibco), L-glutamine (10 mM) and HEPES (10 mM). L-glutamine, HEPES and gentamicin were from Sigma.

### Bacterial Infection of Cultured Macrophages

Infection of cells was done by the gentamicin protection assay (Eriksson et al., 2003). For opsonization, a bacterial colony was resuspended in PBS and incubated with 10% pre-immune BALB/c mouse serum for 30 min at 37°C in PBS prior to infection of the cells at a multiplicity of infection (MOI) of 10. After 1 h of phagocytosis, medium containing 50 μg/ml of gentamicin was applied for 45 min to kill extracellular bacteria. Medium containing 10 μg/ml of gentamicin was applied for the rest of the incubation time of infected cells which was up to 18 h, depending on the experiment.

In the experiments using plasmid carrying bacteria, the media was supplemented with 20 mM L-arabinose (Sigma) to induce bacterial expression of GFP. To obtain non-replicating bacteria, tetracycline (10 μg/ml, Sigma) was added, from 2 h p.i. throughout the duration of the experiment, to block bacterial protein synthesis (Negrea et al., 2007).

When stated, the cells were pre-stimulated overnight with lipopolysaccharides (LPS) from *E. coli* (Sigma; 5 to 5,000 ng/ml) or peptidoglycan from *Staphylococcus aureus* (Sigma; 30 μg/ml).

### Detection of Hypoxia

2-Nitroimidazole compounds form complexes with thiols under hypoxic conditions (Varghese et al., 1976). To detect hypoxia, the cells were treated with pimonidazole hydrochloride (hypoxyprobe-1;NPI, Inc., Belmont, MA, USA) at a final concentration of 100 μM for 2 h prior to fluorescence labelling and microscopy to detect bound pimonidazole.

### Immunofluorescence Staining

Cells were seeded on glass coverslips 24 h prior to infection. At indicated times after infection, the cells were washed with PBS and fixed in phosphate-buffered 4% formaldehyde (pH 7.2) for 10 min at room temperature. Fixed cells were washed three times with PBS, and incubated in blocking buffer, 10% FBS with 0.2% saponin, for 20 min at room temperature. Primary antibodies were rabbit anti-iNOS polyclonal (1:400; Santa Cruz Biotechnology, Santa Cruz, CA) and mouse anti-pimonidazole monoclonal IgG1 (1:200; hypoxyprobe-1;NPI, Inc., Belmont, MA, USA). Secondary antibodies were Cy3-conjugated goat anti-rabbit IgG and Dylight405-conjugated goat anti-mouse IgG (1:25; Jackson ImmunoResearch, Suffolk, UK). Antibodies were diluted in blocking buffer and added to the cells for 45 min. DNA was stained with DAPI (1:1,000) for 5 min. Stained coverslips were then mounted on microscopy slides and observed under an upright fluorescence microscope (Nikon Eclipse TI) or an inverted confocal microscope (Zeiss LSM700).

To detect protein synthesis, cells were infected and fixed following treatment with protein synthesis inhibitor puromycin at different time points as described previously (Panas et al., 2015). Briefly, before fixation, the growth medium was replaced with medium containing 10 μg/ml of puromycin and incubated for 5 min at 37°C. The cells were then washed twice with PBS followed by fixation using 4% paraformaldehyde in PBS for 10 min at room temperature. Permeabilization was performed using methanol for 10 min at -20°C followed by two PBS washes and addition of blocking buffer (5% horse serum, Sigma, in PBS) for an overnight incubation at 4°C. Primary antibody was anti-puromycin antibody (MABE343 from Millipore, 1:500). Secondary antibody was Alexa Fluor 555 (Molecular Probes, 1:500) and Hoechst stain (1:1,000). The cells in the puromycin experiment were imaged using a Supercontinuum Confocal Leica TCS SP5 X microscope with an additional pulsed white light laser and Leica HCX PL Apo 63x/1.40 oil objective.

### Flow Cytometry

Cells were grown in 6 well plates, treated with LPS or peptidoglycan as described above. At the desired time point, the cells were harvested by treatment with Trypsin-EDTA (Gibco; 0.25%) at 37°C, moved to 15 ml Falcon tubes and fixed in 4% formaldehyde for 10 min at room temperature. The cells were incubated in blocking buffer, 10% FBS with 0.2% saponin, for 20 min at room temperature and labeled with the primary antibody rabbit anti-iNOS polyclonal (1:400; Santa Cruz Biotechnology, Santa Cruz, CA) and the secondary antibody Cy3-conjugated goat anti-rabbit IgG (1:25; Jackson ImmunoResearch, Suffolk, UK). The cells were counted using a MACSQuant Analyzer (Miltenyi Biotec). The data was analyzed with FlowJo. The gating strategy are shown in **Figure S1**.

### Statistical Analysis

The *in vitro* infection experiments were performed in triplicate and repeated at least twice. The stained cells were screened for intracellular bacteria, hypoxia and/or iNOS. GraphPad Prism v6.0g (GraphPad Software, Inc., USA) was used to perform statistical tests. The results were analysed by two-tailed Student’s t-test to determine statistical significance between the values for the different groups and a one-way ANOVA, with Dunnett’s correction applied, was performed when comparing multiple groups.

### LDH Assay

The LDH assay was performed using Cytotoxicity Detection KitPLUS (LDH) from Roche. The samples for LDH measurements were obtained by recovering supernatant from uninfected and infected wells at 6 h and 18 h post infection with two biological replicates performed with technical triplicates for each conditions and time point. The values for each time point are normalized to maximal lysis for the specific time point.

## Results

### Intracellular Replication of *S*. Typhimurium Associates With Hypoxia

**Figure 1:**
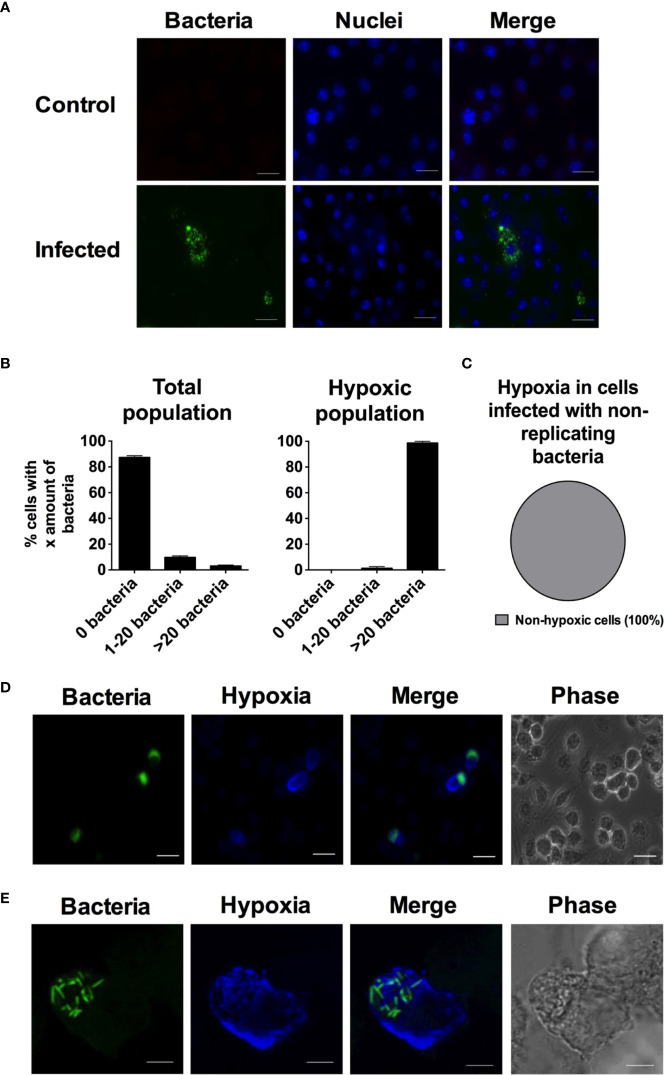
During infection with *S*. Typhimurium the heavily infected macrophages become hypoxic. RAW264.7 macrophages were infected with wild type *S*. Typhimurium expressing YFP. At 14 h p.i. the cells were treated with 100 µM pimonidazole. At 16 h p.i., the monolayers were fixed. In **(A)**, cells were stained with DAPI to detect macrophage nuclei. In **(C)**, infected macrophages were treated with tetracycline from 2 h p.i. to inhibit bacterial replication. In **(D, E)**, the cells were immunostained with anti-pimonidazole antibody for detection of hypoxia. **(A)** Representative images of a monolayer of RAW264.7 cells infected with *S*. Typhimurium and a non-infected control. Green-bacteria; blue-macrophage nuclei. Scale bars, 10 μm. **(B)** The percentage of cells containing 0, 1–20, or >20 bacteria in the total population of macrophages exposed to *S*. Typhimurium or in the hypoxic population of the infected macrophages. The number of intracellular bacteria was counted for 940 cells in the total population and 49 in the hypoxic population. The experiment was repeated three times. **(C)** 5,000 cells from two independent experiments were scored for hypoxia. **(D)** Representative image of an infected and hypoxic RAW264.7 cells. Green-bacteria; blue-hypoxia. Scale bars, 10 μm. **(E)** Confocal image of an infected and hypoxic macrophage. Green-bacteria; blue-hypoxia. Scale bars, 5 μm. For **(D, E)**, the phase is included to show the outline of the individual cells.

*Salmonella* seeks to and replicates in necrotic tumor tissue, likely representing a hypoxic environment (Forbes et al., 2003). We hence wanted to determine if and how hypoxia would develop on a single cell level in a cell culture setting. To exploit this, un-primed murine phagocytic RAW264.7 cells, a cell line that responds to hypoxia (Fong et al., 2007; Srinivasan and Avadhani, 2007) and that expresses HIF (Acosta-Iborra et al., 2009; Liu et al., 2009), were infected with fluorescent *S.* Typhimurium. Hypoxia was detected with pimonidazole fluorescence microscopy 16 h post infection (p.i.). We have previously shown that the uptake of *S.* Typhimurium by RAW264.7 cells is about 10% from an MOI of 10, and with a subsequent growth yield by a factor of ten by 16 h p.i. (Puiac et al., 2009). At this time point, in accordance with previous work on primary BMDM (Saliba et al., 2017) or RAW264.7 cells (Helaine et al., 2010), we detected RAW264.7 cells containing no bacteria, cells with one or a few intracellular bacteria, and cells with a large number of intracellular bacteria (**Figures 1A, B**). Almost 90% of the cells did not contain detectable bacteria, while about 10% contained 1–20 bacteria and only a small percentage of the cells contained more than 20 bacteria (**Figure 1B**). We have previously shown that application of a SPI2 inhibitor, hindering the ability of the bacteria to replicate inside the host cell, removes the heavily infected cells (Puiac et al., 2009), implying that the heterogeneity in bacterial load we observed originated from differential replication inside the cells and not differential phagocytic propensities of the RAW264.7 cells.

We have identified three populations within the infected RAW264.7 cells. We were interested to determine whether hypoxia appeared evenly throughout the three groups of cells; the uninfected cells, the cells containing low numbers of bacteria, and the cells having experienced extensive bacterial replication. Throughout the whole population, the large majority of cells contain no or less than 20 bacteria. When we looked at the hypoxic population, the result was quite the opposite, where no hypoxic cells were uninfected and almost no hypoxic cells contained only 1–20 bacteria (**Figure 1B**). In fact, almost 100% of the hypoxic cells contained over 20 bacteria and had experienced robust bacterial replication (**Figures 1B, D**). **Figure 1E** shows a close-up confocal image of a hypoxic cell, where a large number of bacteria are clearly visible and the entire cytoplasm is stained for hypoxia. To further establish if development of hypoxia was dependent on bacterial replication, we treated the infected cells with tetracycline to stop bacterial replication. We could clearly see that bacterial replication was inhibited as we did not find any RAW264.7 cells containing over 20 bacteria. We scored 5,000 cells and could not detect any hypoxia (**Figure 1C**). The fact that only cells with replicating bacteria turn hypoxic indicates that the replicating bacteria are inducing the hypoxic state, most likely by consuming oxygen while growing.

### *S*. Typhimurium Preferentially Replicates in Cells Lacking iNOS Expression

It is well described that macrophages respond to *Salmonella* infection by producing reactive nitrogen intermediates generated via** iNOS activity (Vazquez-Torres and Fang, 2001). Hypoxia is linked to many parts of the innate immune response of macrophages, for example, the production of NO, since the responsible enzyme iNOS requires oxygen (Vazquez-Torres and Fang, 2001). Furthermore, hypoxia comes with induction of the hypoxia inducible factor HIF that participates as an inducer of iNOS expression (Saliba et al., 2017). We thus wanted to explore the relationship between bacterial infection, hypoxia, and iNOS expression in RAW264.7 cells. As shown previously, inhibition of iNOS results in a decreased NO production and an increased bacterial growth yield (Eriksson et al., 2000). More specifically, we have demonstrated that application of the iNOS inhibitor L-NMMA enhances intracellular replication of *S*. Typhimurium 14028 in RAW264.7 cells, whereas an iNOS activator does the opposite (Puiac et al., 2011). However, the majority of studies on iNOS activity were performed on the total population of cells.

**Figure 2:**
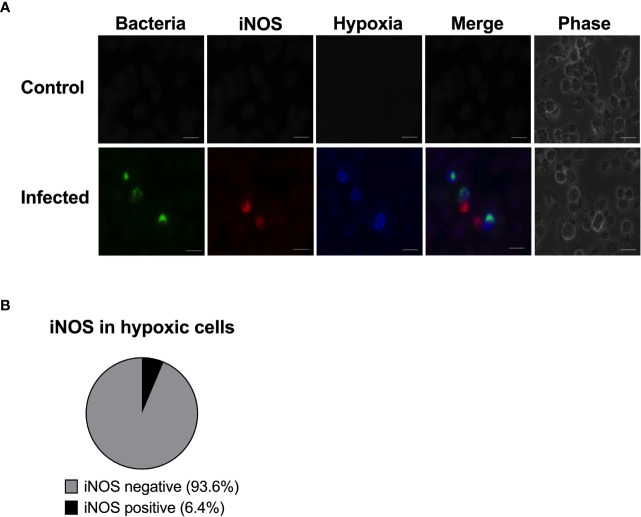
The hypoxic cells are almost never iNOS positive. RAW264.7 macrophages were infected with wild type *S*. Typhimurium expressing YFP. At 14 h p.i., the cells were treated with 100 µM pimonidazole. At 16 h p.i., the monolayers were fixed and immunostained with anti-iNOS antibody to detect iNOS and anti-pimonidazole antibody to detect hypoxia. **(A)** A representative image is shown. Green-bacteria, Red-iNOS; blue-hypoxia. Scale bars, 10 μm. **(B)** 251 hypoxic cells were scored for iNOS activity. The experiment was repeated three times. Error bars are standard error of the mean.

To get an overview of iNOS, hypoxia and replicating bacteria at the single cell level, we infected the RAW264.7 cells with fluorescent *S*. Typhimurium and double-stained for hypoxia and iNOS. A control population of macrophages not exposed to bacteria showed no sign of hypoxia induction or of the iNOS response (**Figure 2A**). We counted 251 hypoxic cells and scored them as either iNOS positive or negative. At 16 h post infection, the majority (94%) of the hypoxic cells were iNOS negative (**Figures 2A, B**). Thus, contrary to our expectation, hypoxia did not associate with iNOS expression in *Salmonella* infected RAW264.7 cells. Instead, it seems that replication of *Salmonella* occurs in iNOS negative but hypoxic cells.

**Figure 3:**
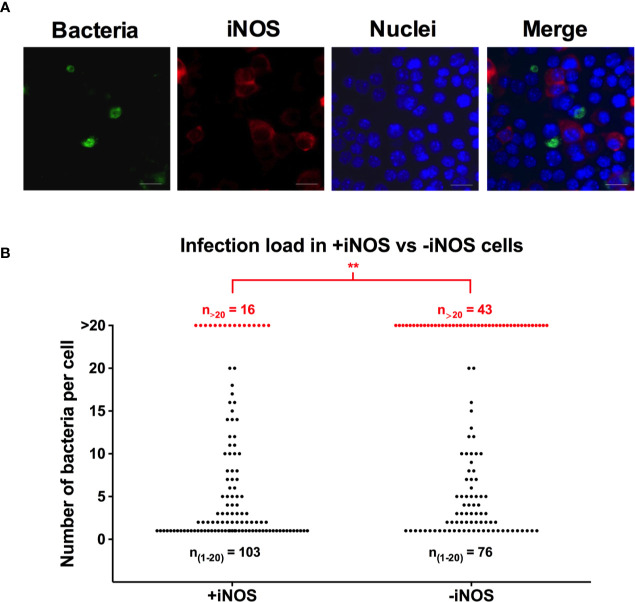
The iNOS positive population of macrophages contains fewer heavily infected cells than the iNOS negative population. RAW264.7 macrophages were infected with YFP-fluorescent *S*. Typhimurium. At 16 h p.i., the monolayers were fixed, and immunostained with anti-iNOS antibody. Cells were stained with DAPI to detect macrophage nuclei. **(A)** Representative image of RAW264.7 cells infected with *S*. Typhimurium is shown. Red-iNOS; green-bacteria; blue-macrophage nuclei. Scale bars, 10 μm. **(B)** Number of intracellular bacteria was counted within the iNOS positive and iNOS negative population of RAW264.7 macrophages. The black dots represent macrophages containing 1–20 intracellular bacteria and the red dots represent macrophages with >20 intracellular bacteria. A two-tailed t-test was used to determine significance between the proportion of cells containing >20 bacteria between the groups with **p < 0.01. The experiment was repeated three times.

To further explore the relation between the replication of *S*. Typhimurium and iNOS expression on the single cell level, we infected RAW264.7 cells with fluorescent *S*. Typhimurium and immunostained for iNOS. Within a population of cells infected with *S*. Typhimurium, we noticed a heterogenic activation of the iNOS response (**Figure 3A**). The proportion of infected cells in the iNOS positive population was 24%, similar to the 20% of infected cells within the iNOS negative population. To quantify the number of intracellular bacteria within cells in the two populations, 119 infected cells were scored within both the iNOS positive and the iNOS negative sub-populations, and the intracellular bacteria were counted. In this, we saw that the macrophages having experienced extensive bacterial replication and containing many, above 20, bacteria there were significantly fewer iNOS positive than iNOS negative cells at 16 h p.i. with 13% of the iNOS positive population (16 out of 119) and 36% of the iNOS negative population (43 out of 119) containing more than 20 bacteria (**Figure 3B**). This result suggests that either is replicating *Salmonella* turning off iNOS expression or *Salmonella* is only able to replicate in an already iNOS non-responding cell.

### Effects of Toll-like Receptor Ligands on Hypoxia and iNOS Expression

When *Salmonella* is invading macrophages, the bacteria are recognized by pattern recognition receptors, such as Toll-like receptors (TLRs), which bind to conserved molecules on the microorganism (reviewed in Rogers et al., 2008). The major TLRs responsible for recognizing parts of the bacterial cell wall are, TLR4, considered the major receptor for Gram-negative bacteria recognizing lipopolysaccharide (LPS) (Chow et al., 1999) and TLR2 forming complexes with TLR1 and TLR6 when responding to peptidoglycan (Takeuchi et al., 1999). The macrophages respond to the invading pathogen by releasing pro-inflammatory cytokines.

**Figure 4:**
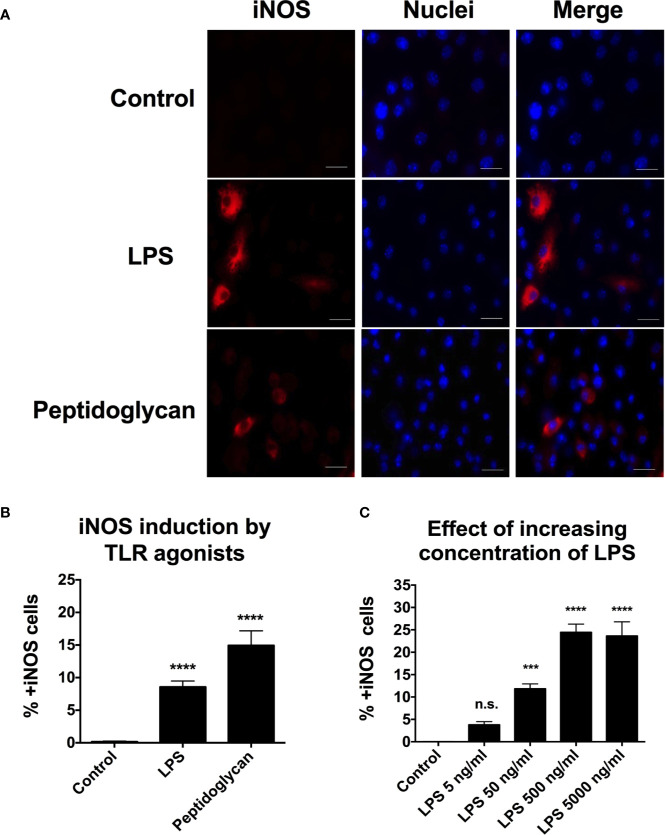
Expression of iNOS is seen after induction with LPS and peptidoglycan but the response is heterogenous throughout the population of macrophages. **(A, B)** RAW264.7 macrophages were incubated in the presence of the LPS (50 ng/ml) or peptidoglycan (30 μg/ml) for 16 h. **(C)** RAW264.7 macrophages were incubated, for 16 h, in the presence of increasing levels of LPS, 5 to 5,000 ng/ml. **(A)** Representative images are shown. Monolayers were fixed, and immunostained with anti-iNOS antibody to detect iNOS. Macrophage nuclei were detected with DAPI. Red-iNOS; blue-macrophage nuclei. **(B, C)** Cells were trypsinized, fixed, and immunostained with anti-iNOS antibody to detect iNOS activity. 100,000 cells were counted by flow cytometry. Data from three independent experiments were combined. A one-way ANOVA with Dunnett’s correction was used to determine statistical significance with ***p < 0.001 and ****p < 0.0001 and n.s, not significant. Error bars are standard error of the mean.

We wanted to use the TLR ligands to understand whether or not bacterial replication is required for the activation of hypoxia and/or iNOS activity. To determine whether TLR agonists in the absence of bacteria, induce hypoxia, and/or iNOS expression, we exposed RAW264.7 cells to purified LPS or peptidoglycan for 16 h. To detect hypoxia, we incubated the cells in the presence of pimonidazole from 14 to 16 h p.i., fixed the cells and immunostained with anti-pimonidazole antibody. We scored at least 5,000 cells for each condition by fluorescence microscopy. To detect iNOS expression, we fixed the cell population and immunostained with anti-iNOS antibody. To quantify the percentage of cells turning on the iNOS response, we stained the treated population of macrophages with anti-iNOS antibody and counted 100,000 cells by flow cytometry. The gating strategy for the flow cytometry can be found in **Figure S1**. Both TLR agonists, without any bacteria present, induced expression of iNOS (**Figures 4A, B**). Similar to the iNOS response activated by infecting bacteria, only a sub-population of the exposed RAW264.7 cells activated their iNOS response (**Figures 4A, B**). In contrast, exposure to the TLR agonists did not induce any detectable hypoxia (**Figure S2**), while the control, infected with *S*. Typhimurium showed induction of some hypoxic cells as above. This result goes well in line with the findings in **Figure 1**, suggesting that bacterial replication is required for induction of hypoxia.

Next, we were interested to know whether the heterogeneity of iNOS induction was dependent on the dose of LPS. We quantified the percentage of cells turning on the iNOS response as a function of increasing concentrations of LPS from 5 to 5,000 ng/ml. We immunostained with anti-iNOS antibody and counted 100,000 cells per condition by flow cytometry (**Figure 4C**). Between 5 and 500 ng/ml, we noticed a dose-related increase in iNOS positive cells. However, there was no further increase in iNOS positive cells between 500 and 5,000 ng/ml of LPS. About 25% of the cells turned iNOS positive even at 5,000 ng/ml (**Figure 4C**), indicating that a proportion of the RAW264.7 cells was unable to respond by expressing iNOS.

These results indicate that the bacteria themselves are not needed for iNOS activation, but the presence of the TLR ligands, LPS or peptidoglycan, is enough. In some infected cells, preferentially the iNOS negative ones, *Salmonella* is able to replicate to large numbers. This heavy replication in turn, uses all the oxygen, turning the cell hypoxic. A final question is whether the iNOS activity is turned off by the replicating *Salmonella*, facilitating extensive replication.

### Late Shut-off of Protein Synthesis in RAW264.7 Cells Infected With *S*. Typhimurium

**Figure 5:**
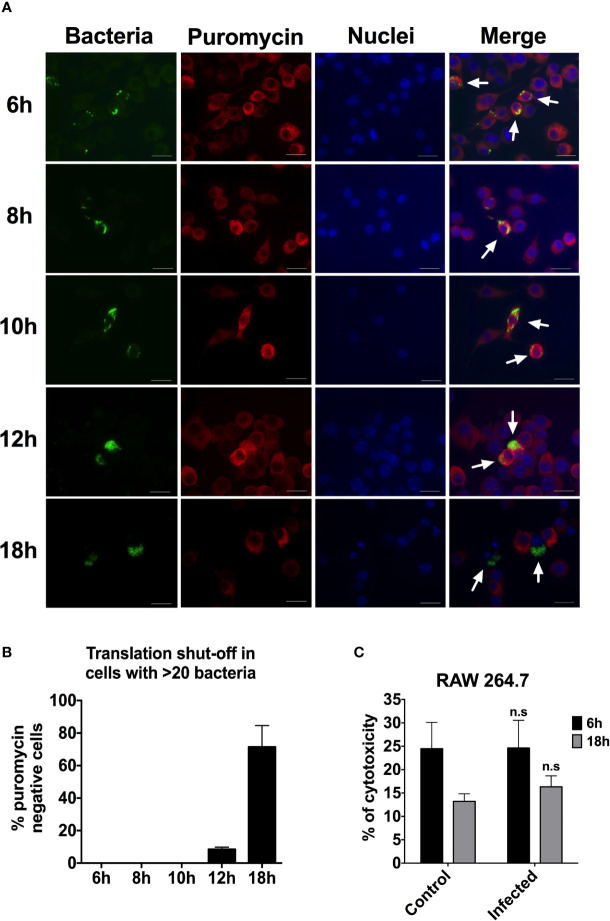
Protein synthesis is beginning to shut down between 12 and 18 h p.i. in RAW264.7 cells infected with *S*. Typhimurium. **(A)** RAW264.7 cells were infected with *S*. Typhimurium expressing YFP. At different time points after infection, the cells were fixed and immunostained with anti-puromycin antibody to detect protein synthesis. The macrophage nuclei were detected with Hoechst. Arrows point to infected cells. Red-puromycin; green-bacteria; blue-macrophage nuclei. Representative images are shown. **(B)** At least 200 cells for each time point were scored for puromycin activity. The percentage of puromycin negative cells, translationally inactive, within the cells with >20 intracellular bacteria are shown. Data are from two independent experiments. **(C)** LDH release 6 and 18 h after infection with *S*. Typhimurium and an uninfected control. A two-tailed t-test was used to determine statistical significance between the control and the infected sample with n.s, not significant. Error bars are standard error of the mean.

Protein synthesis has been shown to be inhibited by growth of invading bacteria, such as *S*. Typhimurium (Koo et al., 1984) and *Legionella pneumophilia* (Moss et al., 2019). We were interested to determine whether replicating bacteria are shutting off protein synthesis in the RAW264.7 cells and thereby inhibit iNOS activity. To test whether bacterial replication prevented protein synthesis, we probed the infected RAW264.7 cell cultures for translation activity on the level of individual cells using the ribopuromycylation staining method. Puromycin binds to the translating ribosome and is incorporated into nascent peptides, whereupon bound puromycin can be visualized using anti-puromycin antibodies and immunofluorescence microscopy. In the absence of translation, no puromycylated peptides will be produced. Time points were taken and immunostained for puromycin activity at 6, 8, 10, 12, and 18 h after infection with *S*. Typhimurium (**Figure 5A**). we quantified the images by counting at least 200 cells for each time point and scoring the puromycin activity. We found no cells containing 1–20 bacteria that were puromycin negative. Within the population of RAW264.7 cells containing more than 20 bacteria and having experienced extensive replication, there were no puromycin negative cells at 6, 8, or 10 h p.i. (**Figure 5B**). At 12 h p.i., 8.5% of the cells containing more than 20 bacteria were puromycin negative and no longer translationally active. This shows that there is no extensive shut-off of translation in the population of macrophages up until 12 h p.i. In contrast, at 18 h p.i., as much as 71.5% of the cells containing more than 20 bacteria were puromycin negative (**Figure 5B**), suggesting a widespread shut-off of protein synthesis. However, at the 18 h time point, only in a few instances did we note nuclear granulation in infected cells, evidencing apoptosis. In parallel, when probing for cell death through release of lactate dehydrogenase (LDH), we could not detect any increase in released LDH activity between a 6 and 18 h post infection (**Figure 5C**). Even if more cells have shut-off their protein synthesis at 18 h p.i., there is no evidence of extensive cell death.

We have determined that at some point between 12 and 18 h p.i., bacterial replication leads to loss of protein synthesis for the majority of infected cells. We performed a time-point experiment to examine the kinetics of iNOS induction after infection. We could clearly detect iNOS expression already at 4 h p.i. (**Figure S3**). The fact that protein synthesis still occurred even in heavily infected cells at 12 h p.i., shows that intracellularly replicating *S.* Typhimurium does not prevent iNOS expression through a general shut-off of protein synthesis. These data also demonstrate that intracellular *Salmonella* and host cell protein synthesis co-existed for at least 12 h.

Taken together, the results of this study suggest that only some cells in a population of macrophages are able to turn on their iNOS at a specific time. Should *Salmonella* infect an iNOS positive cell, the bacteria will be less likely able to replicate extensively. However, if *Salmonella* infects an iNOS negative cell, the bacteria will be more likely able to replicate to large numbers. At later times during the infection, the cells experiencing extensive bacterial replication becomes hypoxic and many cells shut down their protein synthesis.

## Discussion

Hypoxia is associated with several pathological conditions and comes with the activation of the eukaryotic transcription factor, HIF (Zinkernagel et al., 2007). With regard to phagocytes, HIF is also activated after exposure of cells to several different bacterial pathogens (Peyssonnaux et al., 2005) to become involved in regulating numerous parts of the innate immune response, including expression of nitric oxide (NO) through inducible nitric oxide synthase (iNOS) activity and production of antimicrobial peptides (Zinkernagel et al., 2007). In addition, HIF suppresses eukaryotic protein synthesis (Liu and Simon, 2004). Intriguingly, NO also stabilizes HIF under normoxia (Hendrickson and Poyton, 2015).

The facultative anaerobe *Salmonella enterica* seems to benefit from both oxidative and hypoxic environments during infection of mammal hosts (Forbes et al., 2003; Jennewein et al., 2015). While in the intestine, *Salmonella* benefit from the oxidative innate immune response by utilizing concomitantly oxidized sulphur compounds as a respiratory electron sink (Winter et al., 2010; Winter and Bäumler, 2011). On the other hand, phagocyte production of reactive oxygen and nitrogen species stand as a central defence mechanism against systemic salmonellosis (Mastroeni et al., 2000). However, in seeming contradiction, *Salmonella* replicates in phagocytes (Monack et al., 2004), wherein exposure to oxidative and nitrosative stress would be apparent.

Here, we have studied the occurrence and distribution of hypoxia, iNOS expression and protein synthesis in *Salmonella* infected murine phagocytic cells using fluorescence microscopy. In accordance with previous reports on primary murine BMDM (Saliba et al., 2017), we noted an uneven distribution of bacterial growth in RAW264.7 cells 16 h post infection with proportions of the cells appearing non-infected, infected with a few bacteria or heavily infected. Hypoxia was clearly detected by fluorescence staining but again the staining was unevenly distributed, strongly correlating with the heavily infected host cell population. Cells staining positive for iNOS appeared already 4 h post infection. Yet, even after 16 h post infection, only a proportion of the RAW264.7 cells became iNOS positive. Interestingly, the majority of cells with high bacterial numbers were iNOS negative.

Macrophages can be activated in different ways, M1 and M2 being the most common states. M1 activity causes tissue damage and inhibits cell proliferation while M2 activity promotes tissue repair and cell proliferation (Mills, 2012). One example of the activities by M1 macrophages is the expression of iNOS (Rath et al., 2014). Our observations regarding an uneven distribution of iNOS expression would be in accordance with a recent transcriptomic profiling of *Salmonella*-infected BMDMs (Saliba et al., 2017), placing replicating bacteria preferentially in cells with an M2 signature. Also, in granulomas generated by *Salmonella*-infection, bacteria persist in M2 polarized macrophages (Pham et al., 2020). Another aspect to the variability in iNOS expression, and hypoxia, could be a heterogenic bacterial gene expression pattern. For example, not all *S*. Typhimurium cells express the SPI1-associated *prgH* gene when grown in rich medium (Hautefort et al., 2003), and not all *S*. Typhimurium cells present inside macrophages express the *spv* virulence genes (Gulig et al., 1993). Thus, the outcome of the macrophage infection may also rely on the amount and the specific transcriptomic signature of the infecting bacteria.

As the MOI increases, the probability of a macrophage being infected with several bacteria increases. A recent study demonstrated that, at higher MOI, invasion deficient *Salmonella* bacteria, that are close to a T3SS-1 dependent invading bacterium, can be taken up in a cooperative manner (Di Martino et al., 2019). In our case we could demonstrate a dose-dependent increase in iNOS expression in RAW264.7 cells in response to increasing concentrations of LPS. However, plain LPS-induced iNOS expression reached a plateau when about a third of the cells became iNOS positive, further pointing to a multi-layered heterogeneity in the host cell response capability.

A further level of heterogeneity in *Salmonella*-phagocyte interaction was recently demonstrated by Huang et al. (2020). While not detailed at the level of individual cells, the study revealed that different serovars of *S. enterica* generated different levels of nitric oxide production and cytotoxicity in avian and bovine macrophages. Notably, while the avian adapted serovar Gallinarum caused high nitric oxide production but low cytotoxicity in avian macrophages, the effect upon infection with *S*. Typhimurium was the opposite.

*L. pneumophilia* was recently shown to down-regulate host cell protein synthesis with the use of secreted virulence-associated proteins (Moss et al., 2019). In our experiments, cells containing large amounts of bacteria tended to be hypoxic and iNOS negative, leading us to explore the possibility that lack of iNOS could be a consequence of global inhibition of protein synthesis. Thus, infected cells were subjected to puromycin staining that reveals active translation (Liu et al., 2012). This staining revealed a robust and comparable protein synthesis whether or not the cells were infected, at least until 12 h p.i. As iNOS expression was detected already at 4 h p.i., a general halt in host cell protein synthesis likely does not contribute to heterogeneity in iNOS expression. Even at 18 h p.i., by far not all of the heavily infected cells revealed evidence for nuclear fragmentation or failure to stain with puromycin. Also, even 18 h p.i., we could not demonstrate any increase in released LDH to implicating any increased apoptosis in infected cell cultures.

In conclusion, our microscopic analyses point to an assorted response of infected cells with regard to hypoxia, iNOS expression, and translation capability in response to *S.* Typhimurium infection. Most notable was the demarcation between hypoxia and iNOS expression, with 94% of the hypoxic cells lacking iNOS expression. As hypoxia induces bactericidal factors in myeloid cells, including RAW264.7 cells (Srinivasan and Avadhani, 2007), the fate of intracellular *Salmonella* could be a race between replication of the bacteria and mounting of killing capacity of the host cell. Gross replication could induce hypoxia, depriving iNOS and phagocyte NADPH oxidase from molecular oxygen. This is supported by the lack of hypoxia induction upon blocking bacterial intracellular replication with tetracycline, or in response to purified LPS or peptidoglycan. This would also be in accordance with the report of Jennewein et al. (2015) demonstrating that macrophages at hypoxic cultivation promote intracellular replication of *S*. Typhimurium. While combatting microbes, the macrophage-derived reactive oxygen species, including NO, act as inducers of apoptosis (Chandra et al., 2000), so also in RAW264.7 cells (Zhang et al., 2005). Thus, generation of a hypoxic microenvironment could not only add to bacterial replication but also to a prolongation in the lifespan of the infected cell.

## Data Availability Statement

All datasets generated for this study are included in the article/**Supplementary Material**.

## Author Contributions

MW, SP, and MR designed the study. MW, SP, KV, NA, and JN performed the experiments. LL and GM provided reagents, tools, and technical assistance for microscopy. MW, SP, KV, and MR wrote the manuscript. All authors contributed to the final manuscript draft. All authors contributed to the article and approved the submitted version.

## Funding

MW was funded by the Swedish Research Council FORMAS Dnr 222-2013-777. SP, KV, NA, and MR were funded by the Swedish Research Council (Vetenskapsrådet) Dnr 4-30 16-2013. MR was also supported as a visiting professor in the Umeå Centre for Microbial Research (UCMR) Linnaeus Program by grant Dnr 349-2007-8673 from Vetenskapsrådet.

## Conflict of Interest

The authors declare that the research was conducted in the absence of any commercial or financial relationships that could be construed as a potential conflict of interest.
