# Supplementary figures and images for: Replication of Salmonella enterica serovar Typhimurium in RAW264.7 Phagocytes Correlates With Hypoxia and Lack of iNOS Expression
Source: Front Cell Infect Microbiol. 2020 Nov 30;10:537782. doi: 10.3389/fcimb.2020.537782 (PMC7734562; doi:10.3389/fcimb.2020.537782)

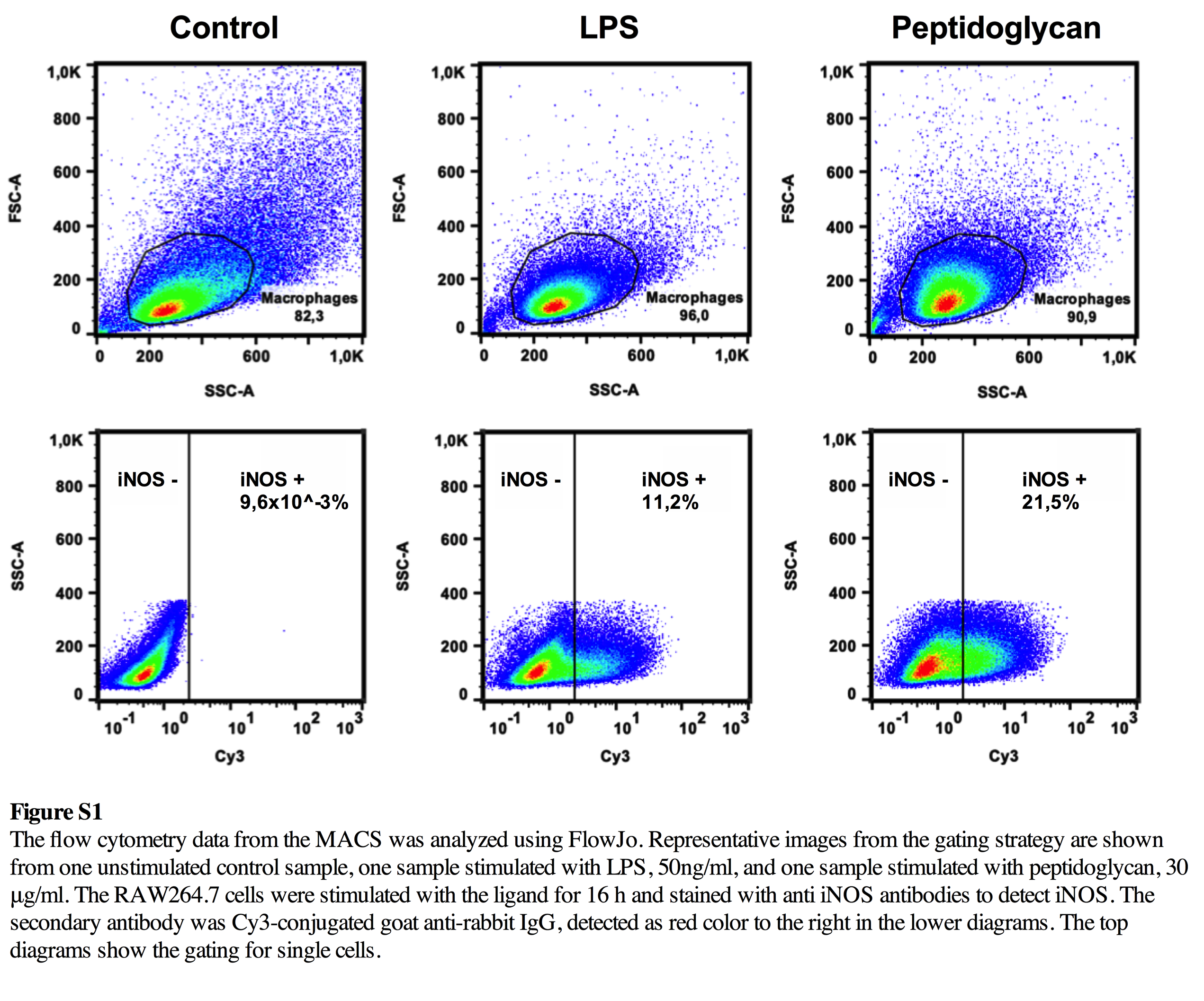

Supplement: Supplementary file 1 [file Image_1.tiff]

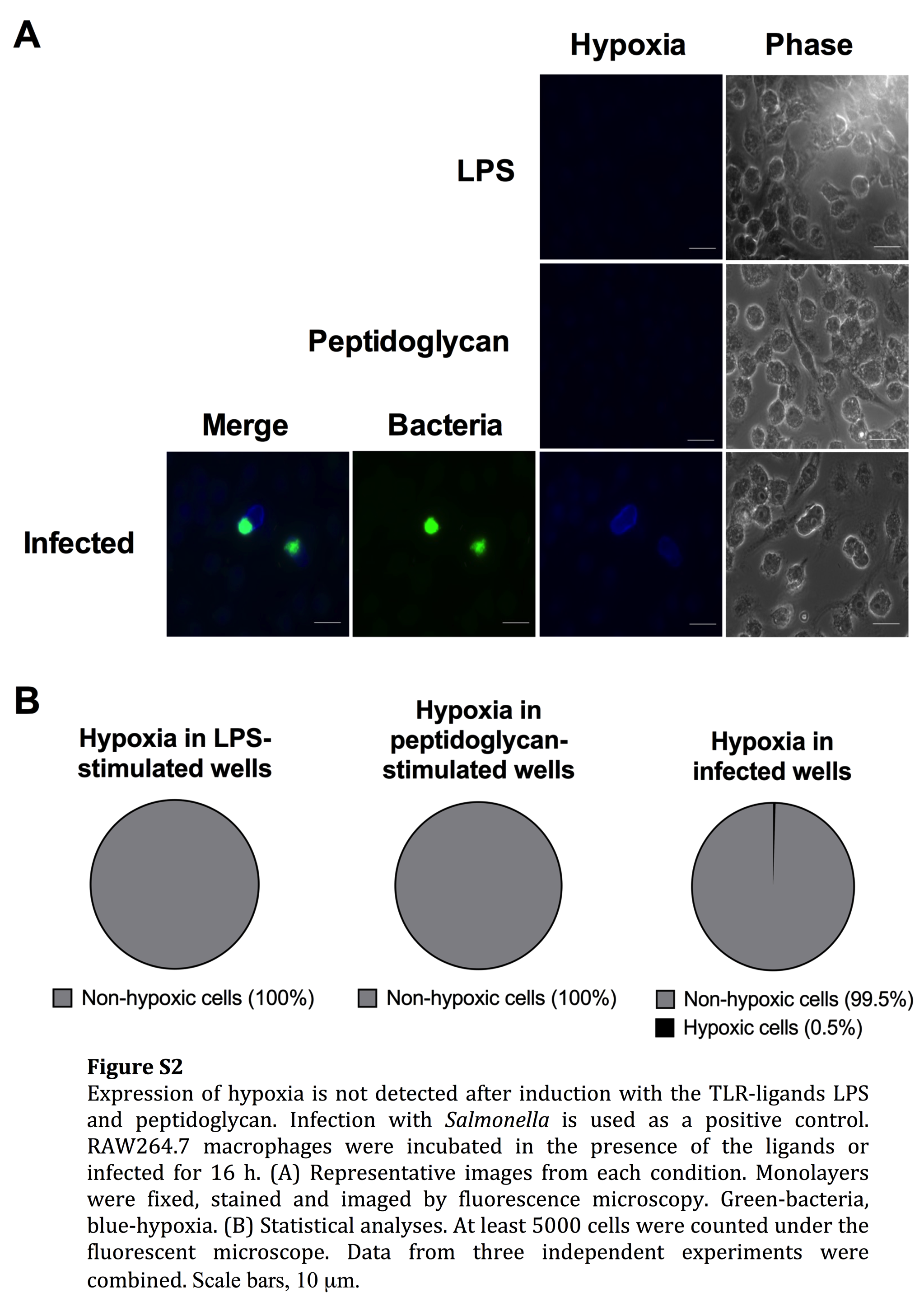

Supplement: Supplementary file 2 [file Image_2.tiff]

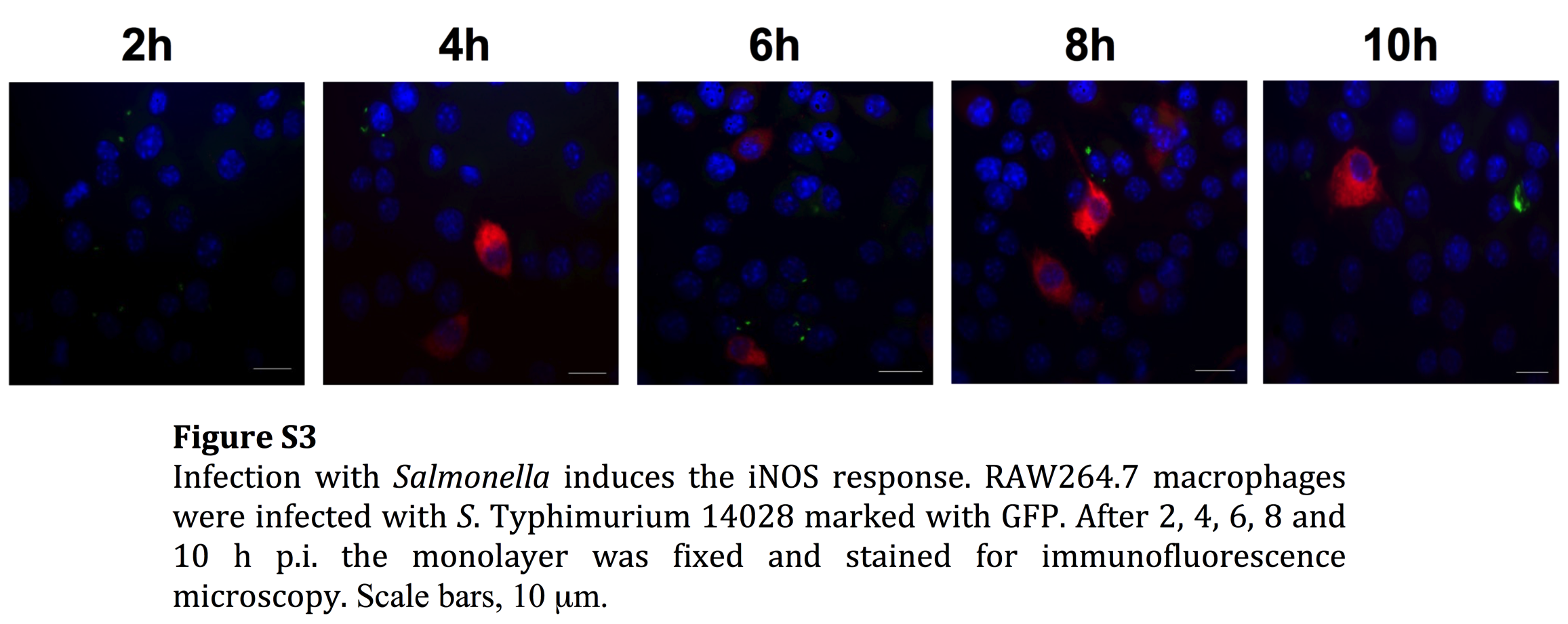

Supplement: Supplementary file 3 [file Image_3.tiff]
